# Sampling SARS-CoV-2 Proteomes for Predicted CD8 T-Cell Epitopes as a Tool for Understanding Immunogenic Breadth and Rational Vaccine Design

**DOI:** 10.3389/fbinf.2021.622992

**Published:** 2021-02-19

**Authors:** Jonathan Hare, David Morrison, Morten Nielsen

**Affiliations:** ^1^ IAVI, New York, NY, United States; ^2^ Bitefirst, Norfolk, United Kingdom; ^3^ Department of Health Technology, Technical University of Denmark, Lyngby, Denmark

**Keywords:** SARS-cov-2, CD8 T-cells, peptides, T-cell vaccine, predicted T-cell epitopes

## Abstract

Predictive models for vaccine design have become a powerful and necessary resource for the expeditiousness design of vaccines to combat the ongoing SARS-CoV-2 global pandemic. Here we use the power of these predicted models to assess the sequence diversity of circulating SARS-CoV-2 proteomes in the context of an individual’s CD8 T-cell immune repertoire to identify potential. defined regions of immunogenicity. Using this approach of expedited and rational CD8 T-cell vaccine design, it may be possible to develop a therapeutic vaccine candidate with the potential for both global and local coverage.

## Introduction

The COVID-19 pandemic is a worldwide health emergency. The first cases were believed to have occurred earlier than December 2019, and as of the beginning of February there have been more than 103,000,000 cases of SARS-CoV-2 infection have been reported worldwide and in excess of 2,200,000 deaths (Update, ecdc.2021). Vaccine development against SARS-CoV-2 is accelerating, and currently there are more than 130 vaccines in development with some progressing to clinical trials in man (Mullard, 2020). Of these vaccines the majority are for a prophylactic indication with the spike glycoprotein being the preferred antigen target.

The full repertoire of immune responses to COVID-19 in patients is still being evaluated but recent publications indicate a significant role for cell mediated immunity in clearing SARS-CoV-2 and conferring some level of protective immunity (Grifoni et al., 2020). This mirrors observations made for other coronavirus infections, SARS (Li et al., 2008), MERS (Zhao et al., 2017) and other viral infections including Ebola (Sakabe et al., 2018) and Lassa virus (Sullivan et al., 2020). Concurrent with vaccine design and understanding the immune response in patients, significant efforts have been invested in understanding the virus phylogeny as it progresses around the globe to identify key sequence changes that may influence vaccine design. The data from this analysis appears to show that the majority of circulating mutations are neutral or deleterious (van Dorp et al., 2020), although a D614G mutation identified in the spike glycoprotein may cause an increase in susceptibility to infection (Korber et al., 2020).

Linking novel sequence data to the use of existing predictive *in silico* tools for epitope identification offers an intriguing approach that can complement existing vaccine design strategies. Previously we hypothesized that incorporating potential immune recognition information into established models may increase the likelihood of success. We have shown that within a population, although HLA sequences show high levels of polymorphism, there are conserved, over-represented alleles that can be used as representative of larger allele diversity (Buggert et al., 2012; Hare et al., 2020a).

Previously we have applied NetMHCpan (Nielsen and Andreatta, 2016) as a proxy to identify putative CD8 T-cell epitopes contained within the HIV transmitted founder virus (TFV) identified from the Protocol C clinical cohort of sub Saharan and East Africa. We have shown that it is possible to stratify and rank protein and/or proteome sequences for their contributions of potential T-cell epitopes (McGowan et al., 2020). Here we propose to use the same approach to evaluate a subset of global circulating SARS-CoV-2 sequences and, using the predefined analysis applied to modeling HIV diversity, identify key regions within the SARS-CoV-2 proteome that could be included within a therapeutic T-cell vaccine.

## Methods

For genes from each SARS-CoV-2 virus proteome sequence all 8–11 mer peptides were generated. The binding affinity of each peptide to the HLA alleles described above was predicted using NetMHCpan-4.1.

Binding predictions below the peptide conservation threshold were read into PostgreSQLR for analysis. The evaluated set of predicted binders was determined as peptides that appear in any virus proteomes, above the peptide conservation threshold of 2.1%. First the virus proteome with the largest number of unique predicted binders was identified. Next, the proteome that, when combined with the previously selected proteome gave the highest increase in coverage of all predicted peptide binders was included. This iterative process was repeated until 100% of all predicted T-cell epitopes were accounted for. For comparison, set-building was performed a second time using randomly selected proteomes instead of choosing the proteome that resulted in the greatest increase of peptide coverage. For more information go to dataspace.iavi.org.

## Results

**FIGURE 1:**
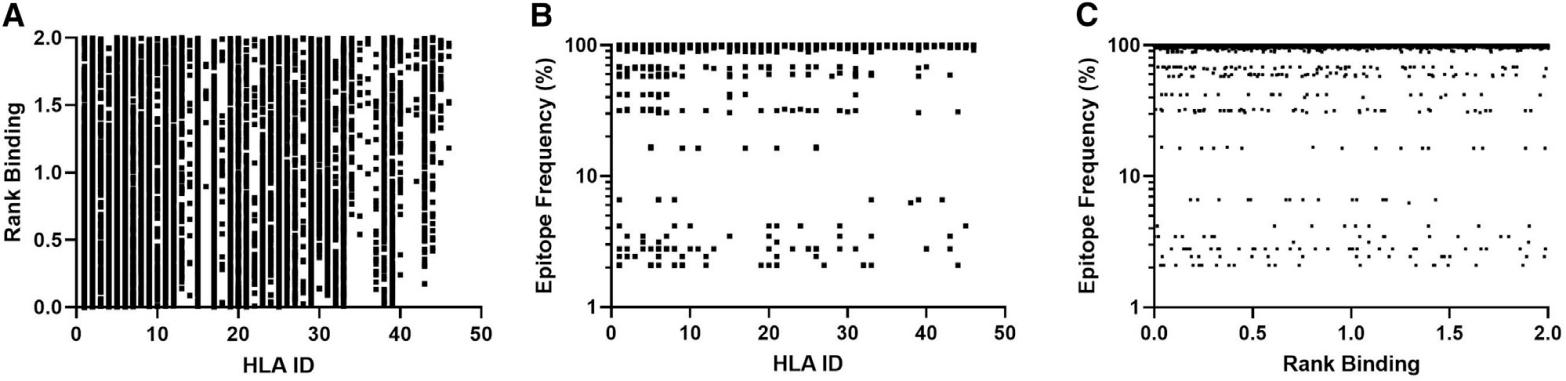
Affinity plots for all predicted peptides with conservation of ≥2.1% (*n* = 11,267). **(A)**–Predicted peptide affinity (Rank Binding) vs. peptide frequency within transmitted founder proteome. **(B)**—Predicted peptide frequency vs. primary associated HLA. **(C)**—Predicted peptide affinity (Rank Binding) vs. primary associated HLA.

Our approach uses NetMHCpan to predict the HLA/peptide binding affinity for the selected virus strains and then identify a set of close binding peptides that are common across the strains. This algorithm was used to analyze 287 SARS-CoV-2 proteome sequences collected between and 08 January and April 02, 2020 and downloaded from NCBI SARS-CoV-2 virus database. The model parameters assessed included a minimum peptide conservation threshold of 2.1% (as previously defined (McGowan et al., 2020)) and a rank binding cut-off of 2.0. Rank binding is used as a metric for peptide:HLA interactions in place of traditional binding affinity scores as it facilitates the normalization of prediction scores across different MHC molecules and enables inter-specific MHC binding prediction comparisons (Reynisson et al., 2020). Figure 1 summarizes the output dataset which includes only those peptides that are present in 6 or more virus proteomes (epitope frequency) and have low rank binding scores although they may represent multiple HLA/peptide interactions (see Table 1 for input sample data and full model parameters).

**TABLE 1:** Model parameters and proteome sources.

Parameter	Values
SARS-COV-2 proteomes	Brazil (1), China (13), France (1), Greece (1), Iran (1), South Africa (1), Spain (10), Sweden (1), Taiwan (1), Turkey (1), USA (238), Unknown (16), Vietnam (2)
Binding threshold	1%
HLA allele contributions	46 alleles (16 x HLA-A, 19 x HLA-B, 11 x HLA-C)
HLA haplotype weighting	None
Rank binding	<2.0
Peptide conservation threshold (%)	2.1%
Peptide length	8, 9, 10, and 11 mers

These frequency and binding thresholds identified 10,982 unique SARS-CoV-2-specific predicted CD8 T-cell epitopes and identified a range of predicted binding profiles for the different peptide-HLA interactions with the majority of peptides associating to selected HLA-A and HLA-B alleles (Figures 1A,B-HLA Ids 1–36) and few primary associations to HLA-C alleles.

**FIGURE 2:**
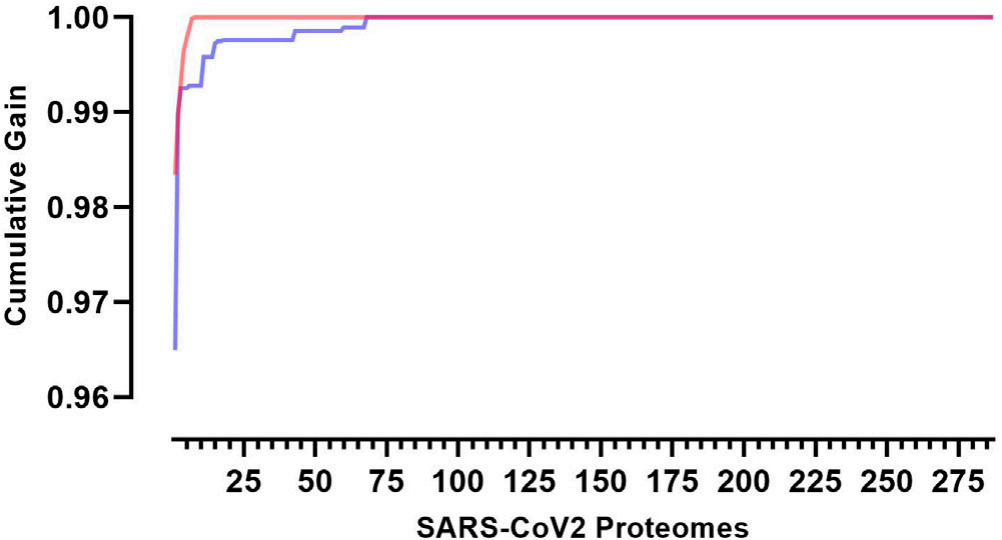
Cumulative coverage distribution plots of full length transmitted founder gag sequences using a 3-select coverage model and a 2% Binding Threshold, 3-Select best (red)) and 3-Select random (blue).

These predicted peptides can be used to assess diversity by assigning a coverage gain value to each sequence. These values can then be used to rank each virus proteome for the coverage it provides within the sample population and by extension identify the sequences that are necessary to obtain the optimum level of epitope restricted sequence coverage (Figure 2).

This model may be used to target and prioritize individual proteomes from which vaccine targets could be derived. In our population of 287 SARS-CoV-2 proteomes each proteome offers an individual coverage gain of 72.2–98.3% against the total landscape of predicted CD8 T-cell epitopes. Using this model eight key proteomes are required to reach 100% epitope coverage within the sample population. It would require an 8-fold increase in virus sequences to achieve 100% coverage if sequences had been selected at random (*n* = 68 *p* < 0.0001).

**FIGURE 3:**
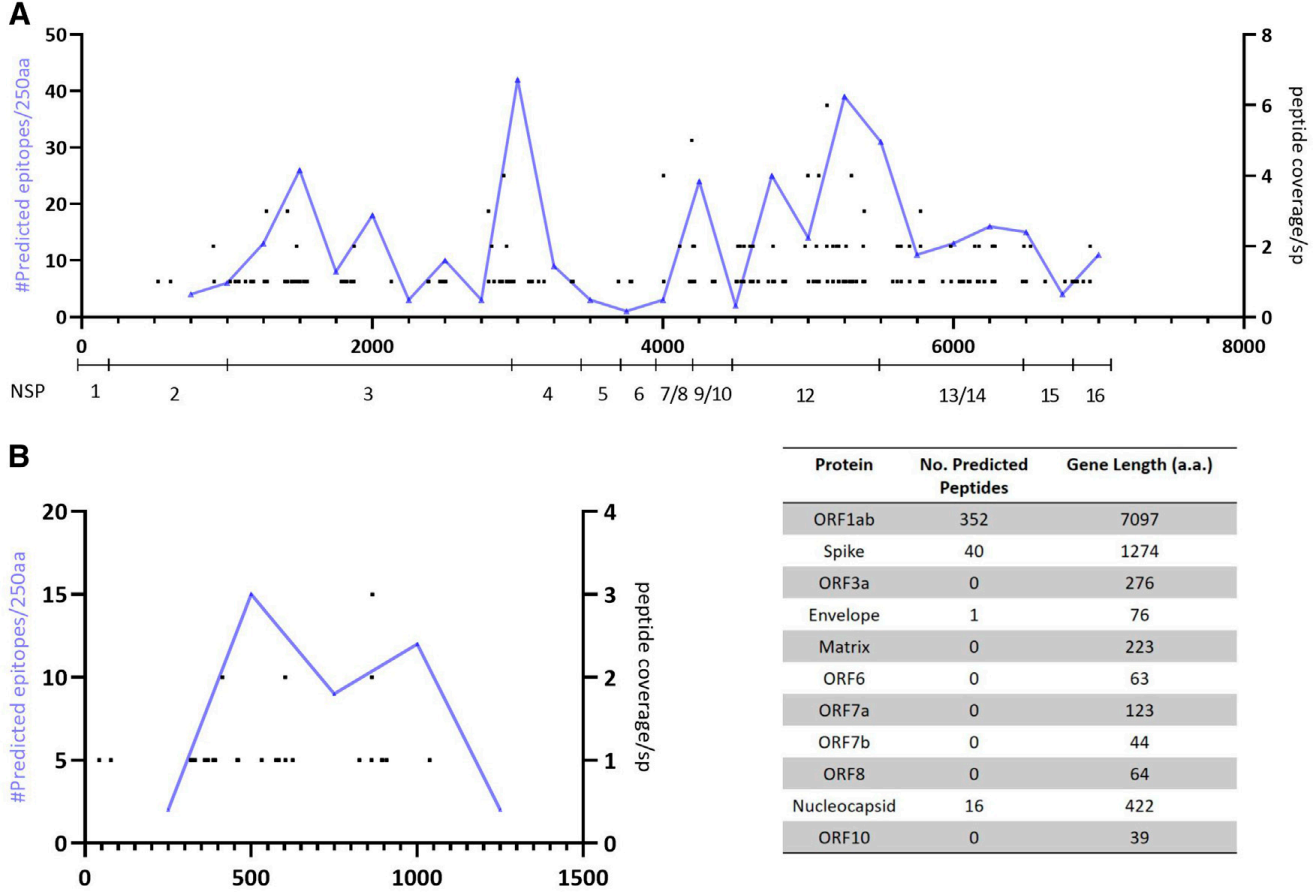
Mapping of predicted epitopes with 100% conservation showing location and number of predicted responses across the proteome and cumulative number of precited epitopes per 250aa. **(A)**—Predicted responses mapped across the ORF1ab polyprotein (with NSP genes identified). **(B)**—Predicted responses mapped across the spike glycoprotein.

Interestingly, of the 10,982 unique predicted CD8 T-cell epitopes (Supplementary Table S1), 409 are contained in all 287 proteomes. These peptides map to four genes within the proteome with 392 peptides contained in ORF1ab polypeptide and spike glycoprotein (Figure 3; Supplementary Table S2).

## Discussion

Evaluating predicted CD8 T-cell epitopes within the SARS-CoV-2 proteome revealed a high level of conservation between proteomes, with each proteome representing 72–98.3% of all predicted T-cell epitopes; however, using our model it is possible to reach 100% epitope coverage using eight defined proteomes. This is in contrast to HIV where it would take 83 proteomes to achieve 100% epitope coverage on a comparable sample size (McGowan et al., 2020). The enhanced conservation of epitopes is in concordance with the observed sequence diversity to date and indicates that no bias has been introduced in to the analysis through modeling with reduced input data.

Moreover, the elevated conservation can be used as a guide to identify regions within the proteome that should be included within a therapeutic T-cell vaccine. Recent data has indicated that naturally occurring T-cell responses in convalescent COVID-19 patients preferentially target the spike glycoprotein and ORF1a polypeptide (Grifoni et al., 2020; Peng et al., 2020). However, limitations in both these studies including that ORF1ab peptides were not examined in one study, the sample size in both was small, peptides were generated from the reference strain and the HLA Class 1 distribution was limited, may mean that potential regions of interest have been overlooked.

We identified 409 predicted CD8 peptides that have 100% conservation within our sample set with >90% of the predicted epitopes contained within either the ORF1ab polypeptide or spike glycoprotein. Furthermore, the predicted epitopes appear to cluster within a ∼550 a. a region of the spike glycoprotein (amino acid 319–865) and within two regions of the ORF1ab polypeptide (amino acid positions 2,750–3,250 and 4,500–5,500). Future experimental testing of these epitopes would confirm whether natural infection induces CD8 T-cell responses targeting these regions, but from an *in silico* perspective they offer a potential target for developing a therapeutic T-cell vaccine that warrants further investigation.

## Data Availability

The datasets presented in this study can be found in online repositories. The names of the repository/repositories and accession number(s) can be found in the article/Supplementary Material.
